# Supplementary material for: The causal role of circulating inflammatory markers in osteoporosis: a bidirectional Mendelian randomized study
Source: Front Immunol. 2024 Jul 18;15:1412298. doi: 10.3389/fimmu.2024.1412298 (PMC11291241; doi:10.3389/fimmu.2024.1412298)
Supplement: Supplementary file 1 [file DataSheet_1.zip › supplementary material/OSTEOPOROSIS-CIM/GCST90274840.tsv.gz_leave_one_out.pdf]

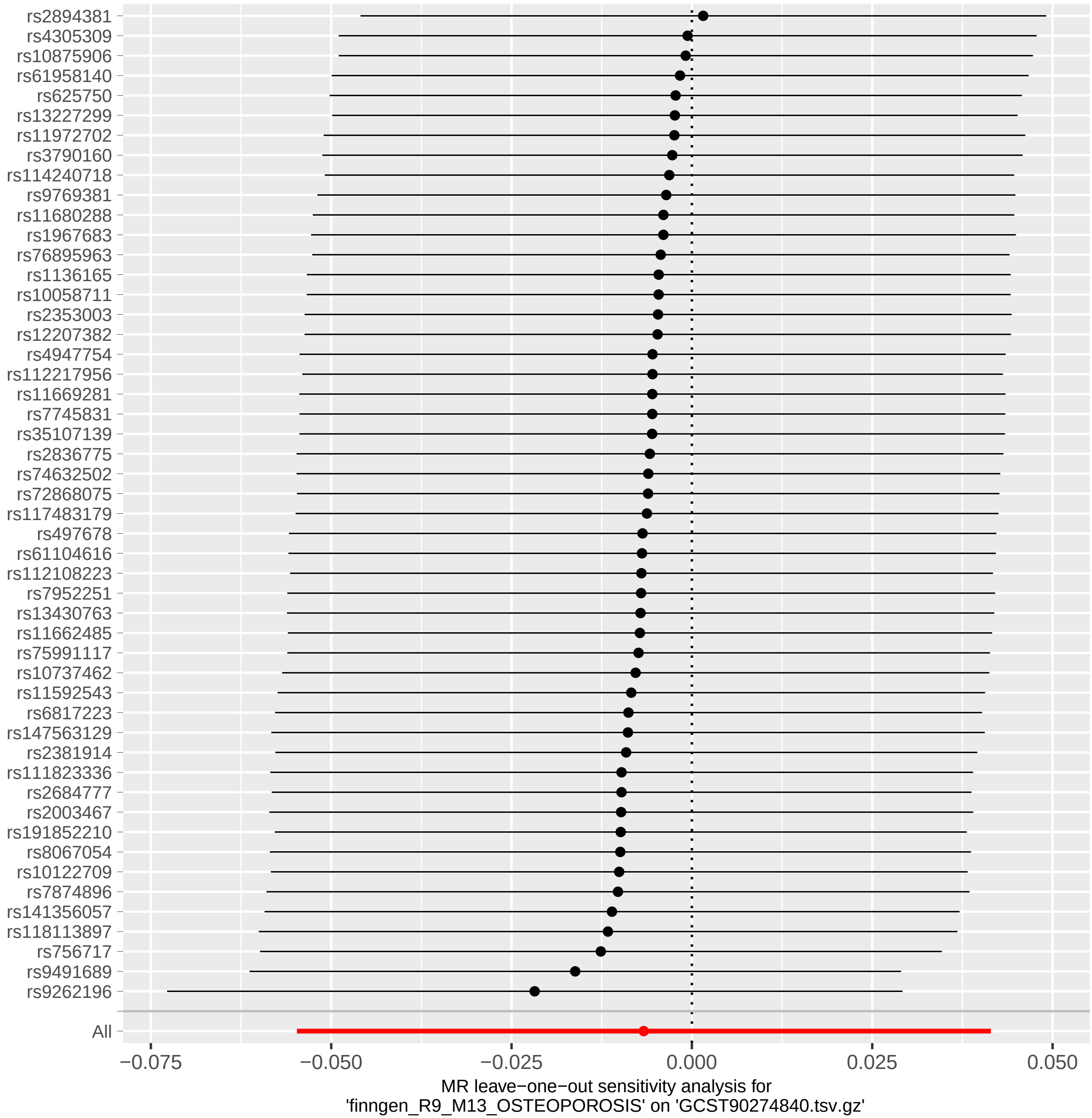
rs2894381
rs4305309
rs10875906
rs61958140
rs625750
rs13227299
rs11972702
rs3790160
rs114240718
rs9769381
rs11680288
rs1967683
rs76895963
rs1136165
rs10058711
rs2353003
rs12207382
rs4947754
rs112217956
rs11669281
rs7745831
rs35107139
rs2836775
rs74632502
rs72868075
rs117483179
rs497678
rs61104616
rs112108223
rs7952251
rs13430763
rs11662485
rs75991117
rs10737462
rs11592543
rs6817223
rs147563129
rs2381914
rs111823336
rs2684777
rs2003467
rs191852210
rs8067054
rs10122709
rs7874896
rs141356057
rs118113897
rs756717
rs9491689
rs9262196
All
−0.075
−0.050
−0.025
0.000
0.025
0.050
MR leave−one−out sensitivity analysis for
'finngen_R9_M13_OSTEOPOROSIS' on 'GCST90274840.tsv.gz'
